# Linking diabetic retinopathy with foot ulcer severity among diabetic patients

**DOI:** 10.6026/973206300211007

**Published:** 2025-05-31

**Authors:** Anunitha R., Sangeetha T., Shasirekha C.A.

**Affiliations:** 1 Department of Ophthalmology, Sri Devaraj URS Medical College, Kolar, Karnataka, India; 2 Department of Surgery, Sri Devaraj URS Medical College, Kolar, Karnataka, India

**Keywords:** Blindness, diabetic retinopathy, foot ulcer, micro-vascular, wagner's grading system

## Abstract

Diabetic retinopathy (DR) and diabetic foot ulcers (DFUs) are common complications of diabetes mellitus, both driven by chronic hyper-glycemia. A significant correlation between Diabetic retinopathy severity and diabetic foot ulcers grade, as well as with elevated HbA1c levels was found among 62 patients in this cross-sectional study. Diabetic retinopathy was classified using the ETDRS system and diabetic foot ulcers by Wagner's grading. The results highlight Diabetic retinopathy as a potential clinical marker for diabetic foot ulcers progression. Integrating retinal evaluation in patients with diabetic foot ulcers may aid in early intervention and improved outcomes.

## Background:

Among the chronic complications of diabetes mellitus, diabetic retinopathy and diabetic foot ulcers (DFU) are particularly significant that impact patient's quality of life and contribute to high healthcare costs [1]. Often resulting from long-term uncontrolled blood glucose, diabetic retinopathy (DR) is a key contributor to blindness in adults of working age which is a key factor that independently is a major contributor to the vascular complications that follow [1]. Diabetic retinopathy progresses in stages, beginning with mild non-proliferative changes involving increased vascular leakage, progressing to severe NPDR marked by vessel blockage and ending in proliferative diabetic retinopathy (PDR), where there is abnormal development of blood vessels on retina, which severely impairs vision [2]. Laser panretinal photocoagulation (PRP), VEGF inhibitors and vitrectomy are treatment options that are used for PDR which can prevent significant vision loss [3]. Diabetic foot ulcers, affecting 15-25% of people with diabetes, stands out as a severe and incapacitating complication that leads to high rates of infection, amputations and hospitalization [4]. These ulcers typically result from a combination of peripheral neuropathy, vascular insufficiency and impaired immune response, making it the leading cause of non - traumatic foot amputations globally [5]. If left untreated, infections from diabetic foot ulcers can lead to limb amputation, significantly impacting patient mobility and independence [6]. Preventive measures, such as regular foot examinations, proper glycaemic control and patient education, are critical in reduce the incidence of diabetic foot ulcers [7]. Both Diabetic retinopathy and diabetic foot ulcers highlight the urgent need for early screening, appropriate management and continuous patient education to prevent irreversible complications in individuals with diabetes. Therefore, it is of interest to investigate and analyse the variation across different clinical stages of diabetic retinopathy and severity grading of diabetic foot ulcers.

## Materials and Methods:

In this cross-sectional study, 62 diabetic patients were enrolled at the Department of Ophthalmology in a tertiary care hospital, between June and September 2024. The study was approved by the Central Ethics Committee (reference number: SDUMC/KLR/R & D/CEC /S/PG/04/2024-25, dated 14/05/2024) and all participants provided written informed consent. All patients with Type 2 diabetes mellitus with Diabetic Foot Ulcer with age more than 18 years were included in the study whereas patients with gestational diabetes, type 1 diabetes < 5 years, Neuropathy, Trauma, Pressure overload, Peripheral artery disease, skin cancer, Osteomyelitis were excluded. All subjects were evaluated for duration, status and medications of diabetes, slit lamp examination was performed to assess the anterior segment and posterior segment was assessed by indirect ophthalmoscopy (IDO). Participants were evaluated for the severity of diabetic retinopathy using the Early Treatment Diabetic Retinopathy Study (ETDRS) classification [12] and for diabetic foot ulcers by Wagner's classification (Grade 0 - Skin intact but bony deformities lead to 'foot at risk'; Grade 1- superficial ulcer; Grade 2 - Deeper, full thickness extension; Grade 3- Deep abscess formation or osteomyelitis; Grade 4 - Partial gangrene of forefoot; Grade 5 - Extensive gangrene) [17]. Additional investigations considered were glycated hemoglobin, blood urea and serum Creatinine. The obtained data was statistically evaluated using SPSS version 22 software with the p-value < 0.05.

## Results:

A total of 62 diabetic patients were included in the study, comprising 38 men (61.3%) and 24 women (38.7%). The average age of the study group was 62.6 ± 11.8 years (range 30-87 years). The duration of diabetes was 7.62 ± 6.9 years. The mean HbA1c level was 9.63 ± 2.49 (range 5.8-16.5). Patient stratification by Diabetic retinopathy severity, as shown in Table 1, revealed that moderate NPDR was the most common stage (29%), followed by no Diabetic retinopathy (21%), mild NPDR (17.7%), severe NPDR (12.9%), early PDR (11.3%), and high-risk PDR (8.1%). Similarly, the categorization of patients based on the grade of diabetic foot ulcers is shown in Table 1. Grade 3 diabetic foot ulcers was the most common, seen in 18 (29%) patients, followed by Grade 1 in 16 (25.8%), Grade 2 in 13 (21%), Grade 0 in 6 (9.7%), Grade 4 in 5 (8.1%), and Grade 5 in 4 (6.5%) of patients. Table 2 shows the statistically significant link between the severity of diabetic retinopathy and different grades of diabetic foot ulcer. Among the 13 participants without DR, 5 had Grade 0, 7 had Grade 1, and 1 had Grade 2 DFU. Among the 11 patients with mild NPDR, 1 had Grade 0, 7 had Grade 1, and 3 had Grade 2 DFU. Among the 18 patients with moderate NPDR, 2 had Grade 1, 8 had Grade 2, and 8 had Grade 3 DFU. Among the 8 severe NPDR cases, 6 had Grade 3 and 2 had Grade 5 DFU. Among the 7 early PDR cases, 1 had Grade 2, 2 had Grade 3, 3 had Grade 4, and 1 had Grade 5 DFU. Lastly, among the 5 high-risk PDR cases, 2 had Grade 3, 2 had Grade 4, and 1 had Grade 5 diabetic foot ulcers (P < 0.001). Although there was a statistically significant difference between diabetic retinopathy grading with respect to HbA1c (P = 0.004, Figure 1 - see PDF), there was no significant association between diabetic retinopathy grading with age or blood urea.

## Discussion:

The increasing prevalence of diabetes has heightened the incidence of complications like Diabetic retinopathy and DFU. Both conditions, if not detected early, can result in irreversible outcomes such as blindness and limb loss. Understanding their association may enable better preventive care. This study, where 61.3% were male and 38.7% were female, with 54.8% having diabetes for less than 10 years and 25.8% for more than 10 years, aligns with recent literature. A study done in 2015 found that 68% of diabetic foot ulcers patients were male, supporting the idea that men are at greater risk for foot complications than women [14]. Another study showed the estrogenic cycle to have a protective effect on neuroretinal function due to estrogens enhancing retinal perfusion through vasodilation. In contrast, testosterone and progesterone may contribute to disease progression by promoting vasoconstriction [15]. The current study data indicates that 25.8% of patients with diabetes exceeding 10 years have a higher prevalence of diabetic retinopathy (DR) and diabetic foot ulcers (DFU), consistent with literature highlighting diabetes duration as a key predictor of these complications. Voigt *et al.* support this, showing Diabetic retinopathy prevalence in type 2 diabetes rises from 12% at 5-10 years to 24% at 10-15 years, emphasizing prolonged diabetes as a major risk factor for Diabetic retinopathy and related microvascular issues like diabetic foot ulcers [16].

79% of patients with diabetic foot ulcers had signs of DR, and 24.4% were diagnosed with proliferative diabetic retinopathy (PDR), which is consistent with a 2023 study that reported a significant association between Diabetic retinopathy and diabetic foot ulcers severity among 100 diabetic patients, with 79% of diabetic foot ulcers patients having Diabetic retinopathy and a notable proportion with PDR (P < 0.001) [18]. Similarly, a meta-analysis reviewed data from 78,529 diabetic patients and found that Diabetic retinopathy significantly increased the risk of developing diabetic foot ulcers (odds ratio = 2.15, P < 0.001) [8]. Another study reported 90% prevalence of Diabetic retinopathy among diabetic foot ulcers patients, with 55% having PDR, and found that factors like older age, higher HbA1c levels, and elevated serum creatinine were linked to diabetic foot ulcers [9]. In the present study, the average HbA1c level was 9.63 ± 2.49% and the average serum creatinine was 1.82 ± 1.24 mg/dL, highlighting the role of poor blood sugar control in these complications. This finding contrasts with a retrospective analysis of chronic foot ulcers and retinopathy progression, which showed 73% of diabetic foot ulcers patients, had PDR [10]. However, the current analysis suggests that most patients with higher-grade diabetic foot ulcers had NPDR changes. Our findings support those of Ramsey *et al.* who identified a strong link between advanced diabetic retinopathy (DR) and poor healing outcomes in diabetic foot ulcers (DFUs). Patients with severe NPDR or PDR were more likely to have higher-grade DFUs, indicating a parallel progression of microvascular complications. This correlation can be attributed to shared pathophysiological mechanisms caused by chronic hyperglycemia. These factors not only hinder wound healing in the lower limbs but also drive retinal ischemia and neovascularization, explaining the frequent co-existence of Diabetic retinopathy and diabetic foot ulcers [11]. A systematic review in 2020 emphasized that patients with diabetic foot ulcers should be monitored by an ophthalmologist and those with Diabetic retinopathy should be promptly referred to a specialized diabetic foot clinic. This suggests that targeted screening in these groups could lead to earlier intervention [12]. Despite the known link between Diabetic retinopathy and DFUs, many patients remain unaware. A recent study titled "An Eye for A Foot: Alarming Unawareness of Diabetic Retinopathy Among Diabetic Foot Patients" found that 50% of patients hospitalized for diabetic foot ulcers (DFUs) were unaware of the risk of diabetic retinopathy (DR) and 80% had never undergone an ophthalmological examination. This highlights a significant gap in patient awareness regarding the ocular complications associated with diabetes [13]. Furthermore, the study "Preventing foot ulcers in patients with diabetes" showed that appropriate management of blood glucose levels could substantially reduce the risk of diabetic foot ulcers, indirectly supporting the interconnection between diabetic retinopathy and diabetic foot complications [7].

## Conclusion:

A strong association between diabetic retinopathy (DR) and diabetic foot ulcers (DFUs) is shown. This supports the need for integrated screening. Proactive screening in high-risk individuals can reduce the burden of complications. Targeted prevention through education and glycemic control remains key.

## Financial support and sponsorship:

Nil

## Figures and Tables

**Table 1:** Characteristics of diabetic retinopathy patients with diabetic foot (N=62)

**Characteristics**		**Frequency**	**Percent**
Gender	Female	24	38.7
	Male	38	61.3
Duration of DM	Newly diagnosed	12	19.4
	<10yrs	34	54.8
	>10yrs	16	25.8
Grades of DR	No DR	13	21
	Mild NPDR	11	17.7
	Moderate NPDR	18	29
	Severe NPDR	8	12.9
	Early PDR	7	11.3
	High risk PDR	5	8.1
Diabetic foot ulcer grading	Grade 0	6	9.7
	Grade 1	16	25.8
	Grade 2	13	21
	Grade 3	18	29
	Grade 4	5	8.1
	Grade 5	4	6.5

**Table 2:** Correlation of diabetic retinopathy & diabetic foot ulcer

**Grading of DR**	**Wagner's grade of Diabetic foot**					
	**Grade 0**	**Grade 1**	**Grade 2**	**Grade 3**	**Grade 4**	**Grade 5**
No Diabetic retinopathy [N =13]	5	7	1	0	0	0
Mild NPDR [N =11]	1	7	3	0	0	0
Moderate NPDR [N =18]	0	2	8	8	0	0
Severe NPDR [N =8]	0	0	0	6	0	2
Early PDR [N =7]	0	0	1	2	3	1
High risk PDR [N =5]	0	0	0	2	2	1
Total	6 (-9.7%)	16 (-25.8%)	13 (21%)	18 (29%)	5 (-8%)	4 (-6.5%)
DR - Diabetic retinopathy, NPDR - Non proliferative diabetic retinopathy, PDR - Proliferative diabetic retinopathy						
